# [^64^Cu]Cu-Labeled αCD11b Diabody as a Novel PET Tracer for the Detection of Immunosuppression in Glioblastoma

**DOI:** 10.1021/acsomega.5c11942

**Published:** 2026-02-19

**Authors:** Bo Li, Sydney A. Jackson, Amelia Stepniak, Peggy Birikorang, Dominic Menendez, Robert Edinger, Michael Pun, Charles M. Laymon, Carolyn J. Anderson, Gary Kohanbash, W. Barry Edwards

**Affiliations:** 1 Department of Biochemistry, 14716 University of Missouri, Columbia, Missouri 65211, United States; 2 Department of Neurological Surgery, University of Pittsburgh School of Medicine, Pittsburgh, Pennsylvania 15224, United States; 3 Department of Radiology, 12317 University of Pittsburgh School of Medicine, Pittsburgh, Pennsylvania 15224, United States; 4 Department of Radiation Oncology, 6614 University of Pittsburgh, Pittsburgh, Pennsylvania 15224, United States; 5 Department of Radiology, 14716 University of Missouri, Columbia, Missouri 65211, United States; 6 Department of Chemistry, 14716 University of Missouri, Columbia, Missouri 65211, United States; 7 Molecular Imaging and Theranostics Center, 14716 University of Missouri, Columbia, Missouri 65211, United States; 8 Ellis Fischel Cancer Center, 14716 University of Missouri, Columbia, Missouri 65211, United States; 9 Department of Bioengineering, 6614 University of Pittsburgh, Pittsburgh, Pennsylvania 15213, United States; 10 Department of Immunology, 6614 University of Pittsburgh, Pittsburgh, Pennsylvania 15224, United States

## Abstract

Glioblastoma multiforme (GBM) is one of the deadliest types of cancer that occurs in people of all ages; 15 months is the average survival time. While treatments for GBM are mostly unsuccessful, immunotherapy has the potential to be an effective strategy for glioblastoma. However, the immunosuppressive influence of tumor-associated myeloid cells (TAMCs) results in poor responses to immunotherapy. As TAMCs are CD11b-positive, the potential of a radiolabeled αCD11b diabody was investigated to assess immunosuppression mediated by TAMCs in an immunocompetent mouse model of glioblastoma. An αCD11b diabody (Db) was constructed with the VH and VL sequences of an αCD11b IgG that resulted in thermal stability and high affinity. αCD11b Db was conjugated with a cross-bridged chelator, CB-TE1K1P, through click chemistry. The resulting conjugate was radiolabeled with ^64^Cu and investigated *in vitro* and in a model of glioblastoma. [^64^Cu]­Cu-αCD11b Db visualized TAMCs in a syngeneic mouse glioblastoma, achieving optimal uptake within 4 h post administration with a %ID/g of 1.06 and 0.18 for tumor and healthy brain tissue. In correlating molar activity (8.51, 4.26, 2.12, and 1.06 MBq/nmol) with uptake (%ID/g of 0.162, 0.825, 1.06, and 0.445, respectively), we demonstrated that 2.12 MBq/nmol gave optimal uptake, since tracer pharmacokinetics was modulated by αCD11b Db occupation of the CD11b antigen sink. In conclusion, [^64^Cu]­Cu-αCD11b Db is a high-affinity and stable diabody, which can quantify CD11b-positive TAMCs in the tumor microenvironment, particularly when the molar activity of the administered [^64^Cu]­Cu-αCD11b Db is optimized for managing the CD11b antigen sink in the spleen, liver, and bone marrow.

## Graphical abstract

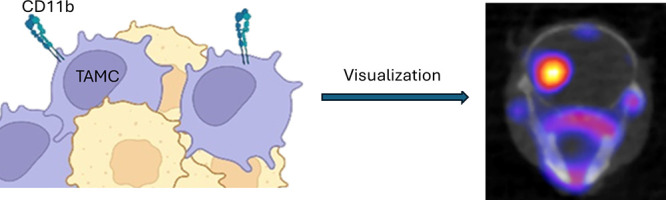

## Introduction

The average survival time for patients with grade IV gliomas (glioblastoma multiforme (GBM)), is 15 months despite treatments such as tumor resection, chemotherapy, and radiation therapy.^1^ While the introduction of checkpoint immunotherapy has been beneficial in other cancer types, such as melanoma, this promising therapeutic strategy has not been successful in GBM. The efficacy is limited by an immune suppressive tumor microenvironment mediated by tumor-associated myeloid cells (TAMCs),^2^ which comprise up to 40% of a GBM’s cellular mass and consist of tumor-associated macrophages (TAMs) and myeloid-derived suppressor cells (MDSCs). In preclinical models, TAMC depletion prolonged the survival of mice bearing syngeneic gliomas.^3^ However, targeting of TAMCs in the clinical setting has had mixed results, in part due to a lack of tools to quantify TAMC infiltration and monitor therapeutic efficacy of TAMC reductions without resection of the tumor.^3,4^ As GBM immunotherapy trials typically continue until disease progression due to a lack of informative biomarkers and access to diagnostic tissue, noninvasive quantification of TAMCs could have a great impact on patient care. Both TAMs and MDSCs overexpress CD11b. While other immune cells, such as eosinophils, also express CD11b, these cells are found in low abundance in gliomas.^5^ Therefore, CD11b is a putative marker of immunosuppression, enabling the immunosuppressive activity of TAMCs to be quantified *in vivo* by CD11b-targeted PET.

We previously demonstrated the CD11b-mediated accumulation of ^89^Zr-DFO-αCD11b (clone M1/70) in a syngeneic model of GBM.^6^ While IgG antibodies generally predominate in immunoPET, smaller antibody fragments, such as diabodies (Db), offer distinct advantages. Due to their lower molecular weight relative to IgG and lack of an Fc domain, diabodies clear more quickly from the blood, which enables potentially shorter imaging times post administration and reduces unwanted immune reactions.^7^ Previous studies showed that the ideal tumor-to-blood imaging ratios with diabody-based radiotracers are achieved within 4 to 6 h postadministration, compared to several days for PET imaging with IgG antibodies.^8^ For these reasons, we have generated an αCD11b diabody by fusing the VH and VL domains sequenced from a hybridoma with a five-amino acid linker, GGGGS, which prevents self-pairing of the VH and VL to form the diabody (Db) as a homodimer. In some instances, diabody conformations will result in instability and loss of affinity that are not suitable for molecular imaging.^9^ Therefore, the central hypothesis addressed is that an αCD11b diabody derived from an αCD11b IgG will be stable, retain high affinity, and localize to CD11b-positive TAMCs in a receptor-specific manner with high contrast within a few hours postinjection.

To address the hypothesis, we expressed the αCD11b diabody in mammalian cells and determined the affinity and thermal stability. We utilized a Cu­(II) chelator, CB-TE1K1P, that provides high *in vivo* stability for good contrast, no trans-chelation to serum proteins, and is well-suited for conjugation to a diabody using click chemistry.^10^ After Cu-64 radiolabeling, clearance was evaluated in an orthotopic syngeneic glioma model with an imaging figure of merit that takes into account radioactive decay to determine the time for optimal tumor-to-blood ratios.^11^ The uptake of [^64^Cu]­Cu-αCD11b Db was dependent on the molar activity, thus demonstrating CD11b-mediated tumor accumulation.

## Results

### Conjugation of CD11b Db with Chelator or Dye

To conjugate a radionuclide chelator to αCD11b Db, an azido-group was conjugated to solvent-exposed lysine residues and N-terminal amines.^12^ The azide motif was then used for the biorthogonal cycloaddition with either the dibenzocyclooctyne (DBCO) of DBCO-PEG4-CB-TE1K1P or AZDye 488 DBCO (Scheme 1). The reason to choose CB-TE1K1P is that the cross-bridge chelator shows very good serum and thermal stability; however, the carboxylic groups of the chelator necessitate conjugation through click chemistry.^10^ As bioorthogonal click reactions are almost stoichiometric, an azide provides a means to determine the average substitution number of the chelators or dyes on αCD11b Db. Therefore, we anticipate that the substitution level of CB-TE1K1P-αCD11b Db can be determined from the substitution level of AZdye488-αCD11b Db, considering that both click reactions are performed with the same azide precursor.

**Scheme 1:**
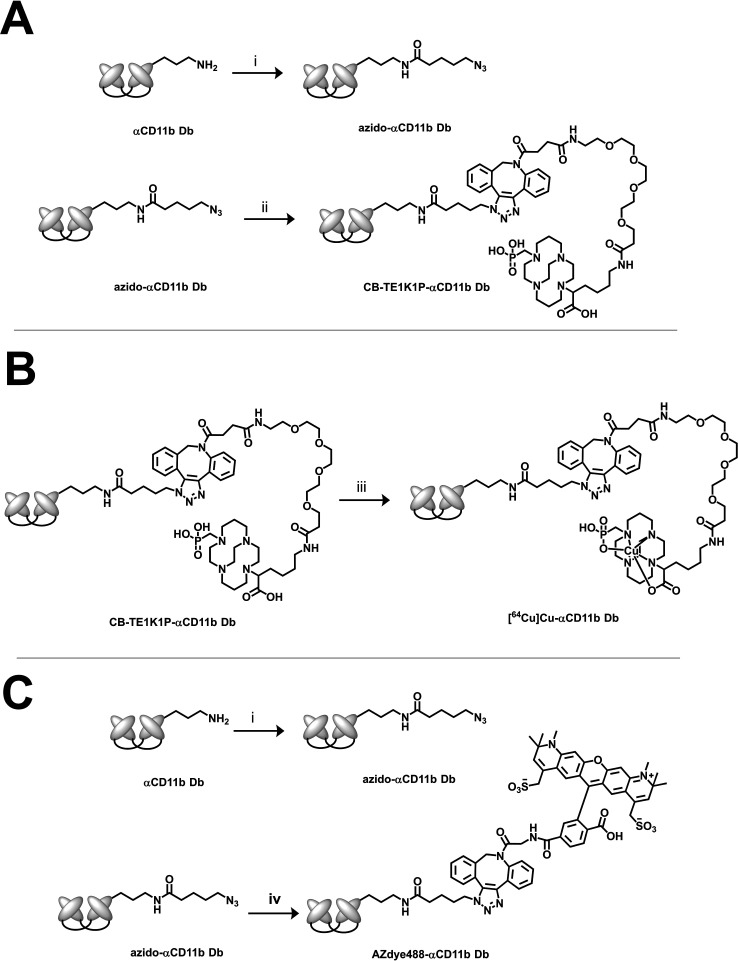
Preparation of Diabody Conjugates (A) CB-TE1K1P-αCD11b Db, (i) Azidobutyric Acid NHS Ester, 37 °C, 1.5 h, (ii) DBCO-PEG4-CB-TE1K1P, 37 °C, 1.5 h; (B) [^64^Cu]­Cu-αCD11b Db, (iii) [^64^Cu]­CuCl_2_, 37 °C, 1.5 h; (C) AZDye488-αCD11b Db, (iv) AZDye 488 DBCO, 37 °C, 1.5 h

Utilizing UV–vis spectroscopy to determine AZdye488 substitution level,^13^ we achieved 1.5 azide:1 diabody with an initial molar ratio of diabody and azide-NHS ester of 1:2.5. Based on this value for the dye substitution level, we assumed the same substitution level for the chelator. CB-TE1K1P-αCD11b Db was then evaluated on an analytical SEC column to determine any induced aggregation, and no aggregation was observed. The retention time (Figure 1) of the conjugate is consistent with the calculated molecular weight (∼53.5 kDa), which was determined with Expasy Protparam.

**Figure 1:**
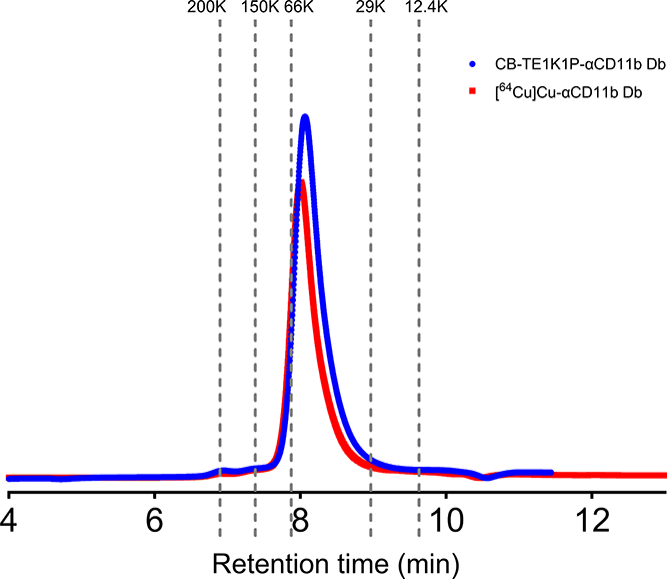
SEC-HPLC chromatogram of the CB-TE1K1P-αCD11b Db and [^64^Cu]­Cu-αCD11b Db radiotracer (PBS, 280 nm).

### Radiolabeling

To radiolabel CB-TE1K1P-αCD11b Db with Copper-64, [^64^Cu]­CuCl_2_ in 0.1 M NH_4_OAc was used as a starting material. Radiolabeling was completed in 2 h with high levels of radionuclide incorporation (radiochemical yield 95%, 8.51 MBq/nmol). The SEC-HPLC chromatograms indicate that the radio conjugate has the correct molecular weight with no apparent aggregation (Figure 1).

### Equilibrium Dissociation Constant Determination

To evaluate the affinity of the αCD11b Db conjugates to CD11b, AZdye488-αCD11b Db was used in flow cytometry experiments with the RAW 264.7 cell line, which expresses CD11b (Figure 2). Fitting the binding data by nonlinear regression determined *K*_d_ = 1.5 nM (95% CI (confidence interval) of 1.1 to 1.9 nM) for αCD11b Db.

**Figure 2:**
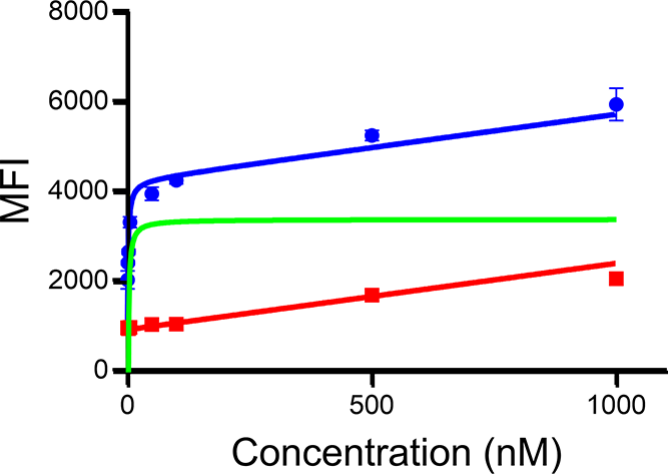
Dissociation constant of AZDye488-αCD11b Db conjugate with RAW 264.7 cell line­(*n* = 3) determined by flow cytometry. Excess αCD11b Db is used to determine nonspecific binding (*n* = 3, *K*_d_= 1.5 nM, 95% CI from 1.1 to 1.9). Blue: total binding; red: nonspecific binding; green: specific binding.

### Immunoreactivity Assay

To determine the binding fraction of [^64^Cu]­Cu-αCD11b Db, an immunoreactivity assay was performed with a CD11b-expressing cell line, RAW 267.4. The cells were stained with either radiotracer only or radiotracer with a 100-fold molar excess of αCD11b IgG. The CPMs (counts per minute) of cell pellets were compared to the CPMs of the radiotracer total added to determine the percent immunoreactivity (Figure 3). The percentage bound of [^64^Cu]­Cu-αCD11b Db was 74%, while for blocking binding, it dropped to 13% for an immunoreactivity of 62% (*t* test: *P* = 0.0006).

**Figure 3:**
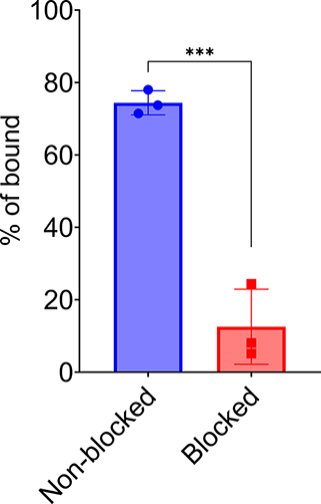
Immunoreactivity assay of [^64^Cu]­Cu-αCD11b Db on RAW 264.7 cells (nonblocked, *n* = 3). Excess αCD11b Db was used to competitively inhibit tracer binding (blocked, *n* = 3). Immunoreactivity is estimated as 61.85%.

### Thermal Stability Assay

To determine the thermal stability of the dye-conjugated diabody, AZdye488-αCD11b Db was incubated at physiological temperatures; then, the binding ability to RAW 264.7 cells was determined by flow cytometry (Figure 4). AZdye488-αCD11b Db that was not thermally treated was used as a reference control. The AZdye488-αCD11b Db at different incubation times was used to stain RAW 264.7 cells, and the mean fluorescence intensity (MFI_test_) was recorded to compare with the MFI of the reference (MFI_reference_, Figure 4). The MFI_test_/ MFI_reference_ at 1, 2, 4, and 24 h were all approximately 100%. Additionally, the inhibition of AZdye488-αCD11b Db binding with excess αCD11b diabody (1000-fold) shows that the observed MFI values are CD11b-mediated. The error bars for the inhibition test are too small to be presented. The results show that the affinity of AZdye488-αCD11b Db is not compromised at physiological temperatures, indicating that there are no thermally induced structural changes that lead to instability.

**Figure 4:**
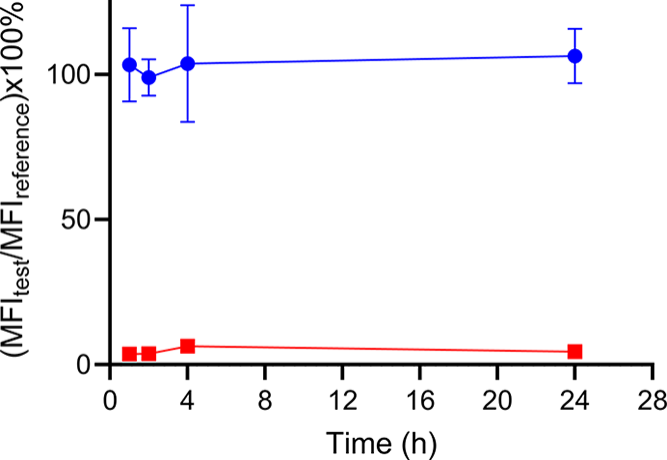
Thermal stability assay of AZDye488-αCD11b Db (*n* = 3) determined by flow cytometry (circles). Excess αCD11b Db was used to competitively inhibit tracer binding (squares).

### PET/CT and Biodistribution

To evaluate the performance of [^64^Cu]­Cu-αCD11b Db for monitoring the accumulation of TAMCs *in vivo*, the radiotracer was evaluated in mice with intracranial GL261 tumors. The effect of antigen sink was demonstrated by varying the molar activity of [^64^Cu]­Cu-αCD11b Db (8.51, 4.26, 2.12, and 1.06 MBq/nmol, Figure 5 and Figure S4). ROI analysis (4 h) of the tissue accumulations was performed by determining the mean standard uptake value (SUV_mean_) of the tumor-specific regions and other CD11b-related organs where macrophages and myeloid cells are found, such as the liver and spleen. The heart was used as a surrogate for the blood. A biodistribution assay was performed after the last imaging time point (24 h, Figure 6). MIPs (maximum intensity projection) are presented in Figures S5 and S6.

**Figure 5:**
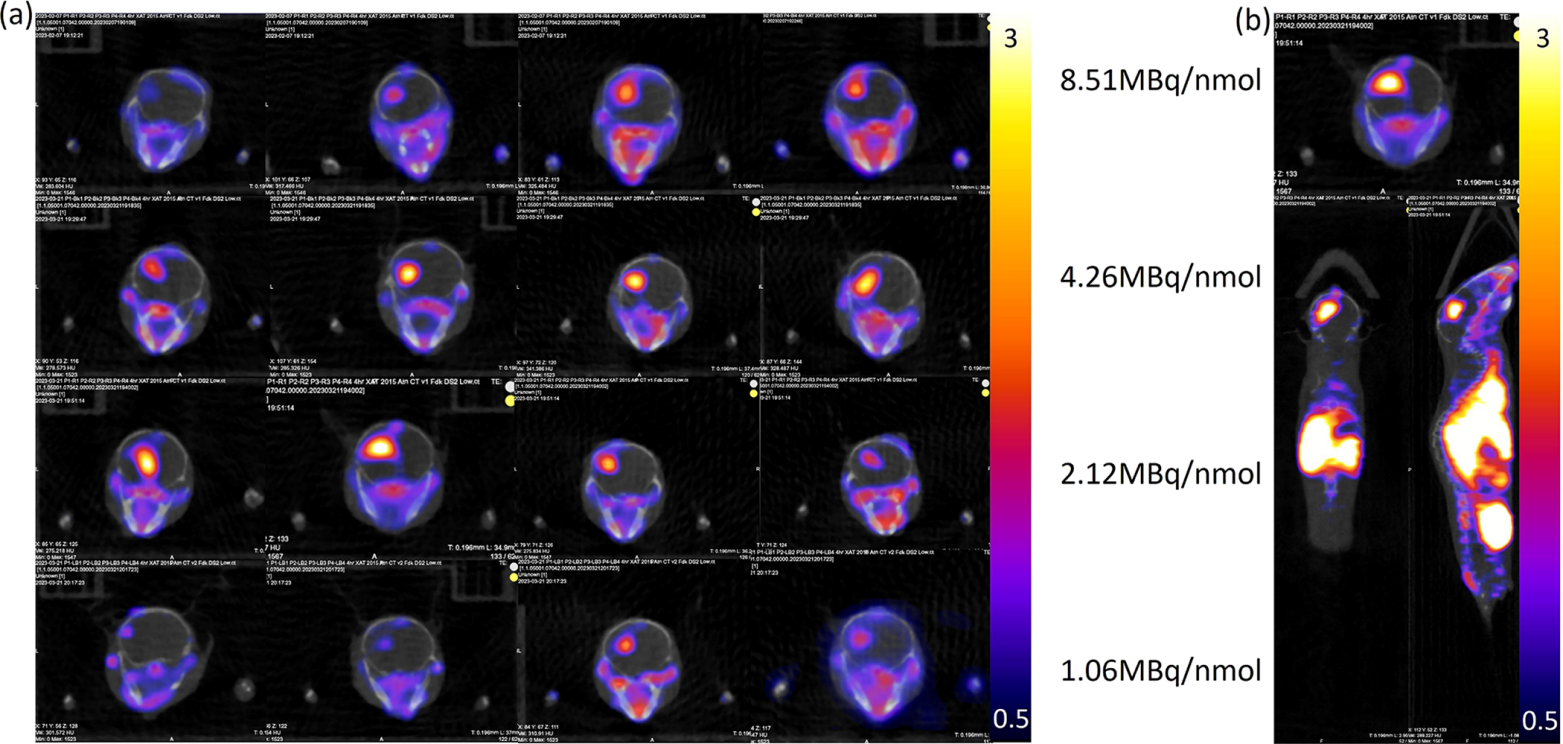
(a) Images of PET scan of mice which received varying molar activity of [^64^Cu]­Cu-αCD11b Db at 4 h (*n* = 4). The color bar represents SUV. The molar activities are 8.51, 4.26, 2.12, and 1.06 MBq/nmol. The total activity injected for each mouse is 3.7 MBq. (b) PET imaging scan of a selected mouse with an axial, coronal, and sagittal view.

**Figure 6:**
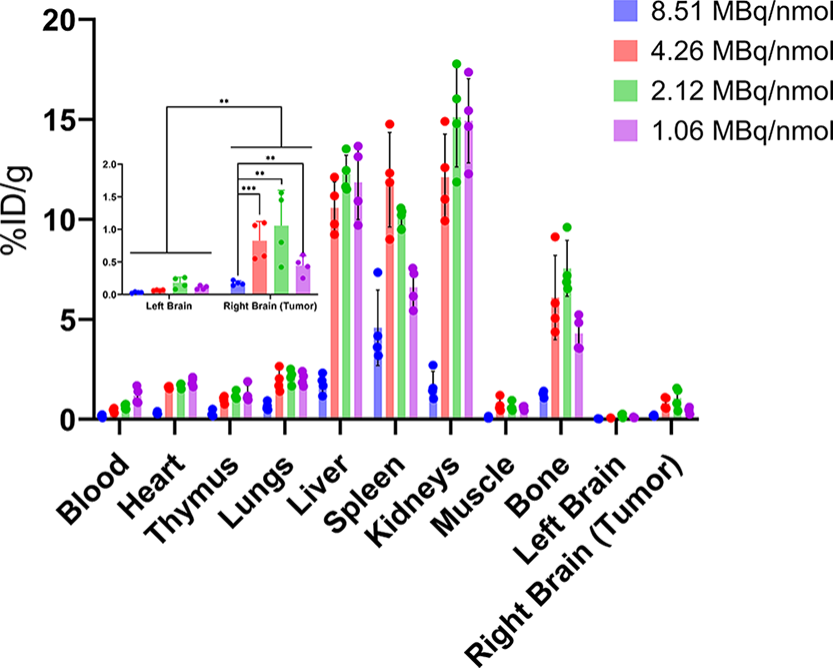
Biodistribution of mice which receive varying molar activities of [^64^Cu]­Cu-αCD11b Db at 24 h postinjection. The molar activities are 8.51, 4.26, 2.12, and 1.06 MBq/nmol. The total activity injected for each mouse is 3.7 MBq. Inset: Detail of the biodistribution of brain and tumor tissue. (*n* = 4).

To demonstrate specific binding of [^64^Cu]­Cu-αCD11b Db to CD11b antigen, the accumulation of the tracer in the tumor and other tissues (SUV_mean_) at 4 h was analyzed at varying degrees of molar activity (Figure S1). SUV_mean_ of tumor and other antigen-rich organs, such as blood^14^ and liver,^15^ changed as the molar activity decreased. There is an apparent maximum of tumor accumulation at molar activities of 4.26 and 2.12 MBq/nmol, but a significant decrease at the minimum molar activity, which is indicative of CD11b-mediated tracer accumulation. This phenomenon has been previously observed.^16^ Apparently, the increased mass of CD11b Db at the lowest molar activity resulted in the inhibition of tumor accumulation. In contrast, at the highest molar activity, the tracer was lost to the antigen sink (Figure S1). These observations were repeated in the biodistribution (Figure 6). We observed that between 2.12 and 4.26 MBq/nmol provided the highest tumor accumulation.

The ratio of SUV_mean_ between tumor and contralateral brain is >5 when the molar activity is 4.26 MBq/nmol. The tumor uptake of the radiotracer significantly differed as unlabeled αCD11b Db was added into the dose (from 8.51 to 1.06 MBq/nmol). Results of SUV_mean,tumor_ show statistical differences between 8.51 MBq/nmol and the other three molar activities (*p* = 0.0007, 0.0062, and 0.0060).

A similar correlation was observed in the biodistribution data (Figure 6), where the group with the highest molar activity (8.51 MBq/nmol) differed from the other three groups with decreasing molar activities (4.26, 2.12, and 1.06 MBq/nmol, *p* = 0.0005, 0.0032, 0.0021, respectively, *t* test). Moreover, in tissues containing CD11b-positive TAMCs (bone marrow, spleen, and thymus), accumulation was blocked by excess αCD11b Db when the molar activity dropped to 1.06 MBq/nmol.

Tracer accumulation changed in CD11b-enriched organs and glioblastoma as the molar activity decreased.^16^ Previous work demonstrates that liver, spleen, and other immune-related organs commonly act as antigen sinks for radiotracers and result in molar activity-induced uptake changes.^14,17^ The statistical difference in the radiotracer uptake among different molar activities in the tumor demonstrates CD11b-specific binding of the radiotracer. Additionally, [^64^Cu]­Cu-αCD11b Db uptake in the contralateral hemisphere (without tumor) was also dependent on molar activity. Possible explanations include the influence of CD11b-positive microglia and/or tumor infiltration into the contralateral side of the brain.^6^ [^64^Cu]­Cu-αCD11b Db uptake in liver, bone, blood, and spleen (Figure S2), which are CD11b-enriched organs (for example, spleen,^18^ liver,^19^ and bone^20^), have a unique accumulation profile with changing molar activity. Both the bone and the spleen display an inverted parabolic relationship with decreasing molar activity. This was not observed with liver and blood, but there are changes in accumulation with molar activity that may be expected for this highly dynamic system.

Although tumor-to-muscle and tumor-to-blood ratios are modest (tumor: blood = 2.0 ± 1.07 and tumor: muscle = 1.27 ± 0.72 at 4.26 MBq/nmol), the tumor-to-brain ratios remain at high values (tumor: brain > 12 at 4.26 MBq/nmol, Figure S3). However, a clinical study shows that the cerebral blood volume of glioblastoma of all stages is no higher than 5%.^21^ Therefore, the small volume of blood within the tumors does not significantly reduce the observed high tumor-to-brain ratio. Finally, the images at 24 h postinjection (Figure S4) show that although [^64^Cu]­Cu-aCD11b Db decreases over time, uptake is retained in the tumor with high contrast between tumor and healthy brain tissue.

### IFOM (Imaging Figure of Merit) Analysis

The traditional figure of merit (FOM) is the decay-corrected ratio of target to nontarget tissue uptake; however, this ratio does not consider the radionuclide half-life. Thus, these ratios continue to increase over time, skewing the perception of the optimal imaging time.^22^ IFOM analysis has been adapted from FOM and takes into account radionuclide decay to guide the selection of an optimal imaging time. In this work, IFOM analysis enabled insight into the earliest time point of maximum contrast between tumor and brain tissue.^11^ This was determined from ROI analysis of the tumor and contralateral brain at 2, 4, 6, 18, and 24 h after radiotracer injection. The higher IFOM means higher tumor uptake of the radiotracer relative to the contralateral brain tissue (Figure 7). The maximum IFOM is at 4 h postinjection­(Figure S7).^23^

**Figure 7:**
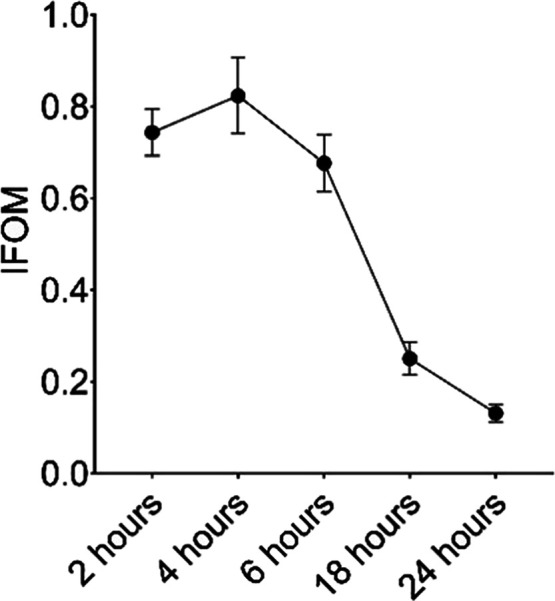
IFOM analysis of [^64^Cu]­Cu-αCD11b Db in GL261 tumors. (*n* = 6, molar activity 8.51 MBq/nmol, 25 μg and 3.7 MBq per mouse, *n* = 6).

Williams et al.^22^ present an imaging figure of merit (IFOM) for selecting an optimum imaging scenario from various given candidates. In their work, they consider the coefficient of variation squared ([CV]^2^) in the net number of counts (tumor counts – background from an equal volume) as a measure of image quality. Their IFOM is taken to be the inverse of the time required to achieve a given CV value. Parenthetically, one possible interpretation of CV is as an inverse signal-to-noise measure.

In IFOM use, the CV represents a minimum required image quality and is set by the observer. As one generally compares IFOM values to identify an optimum imaging scenario, the CV (an overall factor in the IFOM) can be set to 1, as done by Williams et al.^22^ in an example calculation. In the current work, we examine tracer uptake as a function of time from injection so that the values of tumor volume and detection efficiency are also overall constants. The reduced IFOM (r_IFOM_) used in the current work is

$$r_{IFOM}=\frac{{\left[1-\frac{1}{R}\right]}^{2}}{\left[1+\frac{1}{R}\right]}\times u_{SUV}$$

where *u*_SUV_ is the tumor SUV uncorrected for decay, and *R* is the ratio of tumor to background (brain tissue) counts. This is the same as the IFOM by Williams et al.^22^ (with the various overall constants set to 1), with the exception that we replace the percent injected activity per gram of tumor (not corrected for decay, <*U*>) used by Williams et al.^22^ with *u*_SUV_. The relationship between the two is

$$u_{SUVR}=<U>\frac{M\rho }{100}$$

where *M* is the animal mass and ρ is the tumor density.

Our imaging task is to detect tumors in normal brain tissue, so we take our background to be brain rather than blood, as in the work of Williams et al.^22^ Finally, we note that we reject imaging scenarios with *R* < 1 (tumor uptake less than background).

## Discussion

Here, we present the production of a thermally stable, high-affinity diabody, assembled from the VH and VL sequences of an αCD11b IgG (clone M1/70), along with a novel ^64^Cu-CB-TE1K1P-αCD11b Db that visualized CD11b+ TAMCs in GBM in a receptor-specific manner with the highest contrast at 4 h postinjection. The data demonstrate the dependence of tumor accumulation on molar activity. At the highest molar activity, tumor accumulation was low due to losses of the tracer to antigen sink; however, as the molar activity decreased, which means more unlabeled αCD11b Db was coinjected with the radiotracer, tumor accumulation peaked between 2.12 and 4.26MBq/nmol putatively due to saturating CD11b in tissues such as the blood and spleen, and then decreased at 2.12 MBq/nmol due to further blocking of CD11b in the tumor. The optimal molar activity achieves the right balance between competitively inhibiting tracer uptake in target and nontarget tissue with unlabeled substrate. Through IFOM analysis, we showed that the best visualization of the tumor relative to normal brain tissue in this model was obtained at 4 h postinjection. Additionally, even though the chelator was conjugated randomly to solvent-exposed amino groups, the affinity was still high, and immunoreactivity was not compromised. This demonstrates that site-specific chelator conjugation is not necessary for this amino acid sequence despite the fact that there is a Lys residue in CDR2 in the VH domain.

Thermal stability is an important attribute for scFv (single-chain variable fragment)-based radiotracers such as Dbs.^24^ In an IgG antibody, the Fab fragment is stabilized by intermolecular bonding between VH and VL as well as between CH1 and CL, with a smaller contribution between the variable domains of VH/CH1 and VL/CL of each chain. In contrast, the stability of a Db depends on the VH/VL interface alone, similar to the stabilizing mechanism of an scFv.^24^ Previous work has demonstrated that scFvs with reduced thermal stability, despite strong receptor affinity, exhibited very low receptor-mediated tumor accumulation relative to scFvs that were more thermally stable, thus demonstrating that binding affinity alone is insufficient for predicting efficacy of scFv-based molecular imaging agents.^25^ Our results demonstrated that αCD11b Db maintained high binding affinity over extended periods at physiological temperatures with no evidence of thermal instability (Figure 4). Additionally, by comparing tumor-to-blood accumulations with a previously developed IFOM, we found the optimal imaging time was approximately 4 h, indicating that ^64^Cu, with a 12.7 h half-life, is a good fit for PET imaging of Dbs.^7^ Together, these results are indicative of a thermally stable diabody with clearance and target localization that is consistent with previous experimentation with diabodies.^23^

The optimization of the molar activity and imaging time of [^64^Cu]­Cu-αCD11b Db demonstrates that this tracer has the potential to assess the status of immunosuppression by quantifying CD11b-positive TAMCs in the microenvironment of GBM at 4 h postinjection compared to 72 h with a ^89^Zr-labeled-αCD11b IgG antibody.^6^ TAMCs primarily include two cell populations: tumor-associated macrophages (TAMs; including peripheral origin and brain-intrinsic microglia) and myeloid-derived suppressor cells (MDSCs).^26^ TAMCs account for up to 40% of a glioma’s cellular mass and play an important role in tumor progression, immunosuppression, and resistance to immunotherapy, making them a high-priority therapeutic target.^2,27,28^ We have previously demonstrated by immunohistochemistry and flow cytometry analysis that the GL261 orthotopic tumors that CD11b+ immune cells abundantly populate the tumor, compared to normal brain tissue.^6^ TAMs and MDSCs share a common myeloid progenitor cell lineage, with TAMs and MDSCs representing mature and undifferentiated developmental states, respectively. In both humans and mice, TAMs are typically identified as CD11b+CD14+ cells. However, MDSC markers differ in mice and humans as CD11b+Gr1+ and CD11b+CD33+HLA-DR-/low, respectively; however, the common biomarker is CD11b.^29^ Therefore, quantification of CD11b-mediated accumulation is a readout of the TAM and MDSC populations. To the best of our knowledge, there have been no attempts to image these cells in humans with glioma who are being treated with checkpoint immunotherapy, despite the fact that there are multiple clinical trials that may target TAMCs in gliomas (e.g., NCT01790503 and NCT02115074). Therefore, the results of [^64^Cu]­Cu-αCD11b Db suggest that this tracer would enable initial or longitudinal assessment of TAMC levels within gliomas before and during anti-TAMC therapies. Thus, patients who would not respond or become nonresponsive to TAMC therapies, as determined by [^64^Cu]­Cu-αCD11b Db SUV, could be taken off study to receive other therapies. Moreover, the rapid clearance of the diabody is amenable to Cu-64 (*t*_1/2_ = 12.7h) and obviates the need for longer-lived radionuclides such as Zr-89 (*t*_1/2_ = 3.2 d).^30^

[^64^Cu]­Cu-αCD11b Db has been successfully synthesized with a controlled chelator substitution number and high radiolabeling efficiency. The radiotracer shows high thermal stability and affinity, resulting in CD11b-mediated tumor accumulation with the best contrast at 4 h postinjection, demonstrating potential for monitoring anti-TAMC therapies in GBM.

## Materials and Methods

### Reagents

All chemicals were commercially available and used without further purification unless otherwise specified. Aqueous solutions were prepared using ultrapure water (18.2 MΩ cm). All aqueous solutions for HPLC purposes were degassed by a vacuum line before use. Azidobutyric acid NHS ester was purchased from Lumiprobe Corporation. AZDye 488 DBCO was purchased from Click Chemistry Tools. CB-cyclam was purchased from Macrocyclics. DCC, H-d-Lys­(Z)–OH, and DBCO-PEG_4_-NHS were purchased from Chemimpex. All antibodies for flow cytometry were purchased from BioLegend unless otherwise specified. [^64^Cu]­CuCl_2_ was purchased from Madison Cyclotron Lab of the University of Wisconsin. αCD11b Db was prepared by Genscript. αCD11b IgG (M1/70) for animal studies was purchased from InVivoMAb.

GL261 cells were obtained from the NCI DCTD Tumor Repository. RAW 264.7 cells were purchased from ATCC. Gel filtration markers kit for protein molecular weights (12,000–200,000 Da) were purchased from Millipore Sigma (USA) for SEC calibration.

### Instrumentation

Purification of bioconjugates was performed using an Agilent 1260 Infinity HPLC with an SRT-10 SEC-100 1.2 × 300 mm column (Sepax Technologies). Instant thin-layer chromatography (iTLC) was performed with iTLC-SG paper (Agilent, SGI0001), with an Eckert and Ziegler BioScan AR-2000 radio-TLC scanner (Bioscan Inc.), and analyzed using Winscan Radio-TLC software (Bioscan Inc.). Radio SEC-HPLC was performed with an Agilent 1260 Infinity HPLC utilizing a Bio SEC-3, 4.6 mm × 300 mm SEC column (Agilent Technologies). Gamma counting was performed using a PerkinElmer 2470 WIZARD2 Automatic Gamma Counter (Waltham). PET/CT data were analyzed with VivoQuant 2021 (Invicro) and Imalytics 3.0 (Gremse-IT GmbH). Flow cytometry experiments were performed on an LSRFortessa (BD Biosciences), and data were analyzed using FlowJo software (BD Biosciences). UV/vis spectra were obtained on an ND-1000 spectrophotometer (Thermo Scientific). Mass spectrum for the DBCO-PEG4-CB-TE1K1P was obtained on an Agilent 1290 Inifinity II UHPLC with a Zorbax Bonus-RP (2.1 × 50 mm, 1.8 μm) column and coupled to an MSD XT mass analyzer.

### Preparation of CD11b Db

VH and VL sequences were obtained by sequencing the CD11b hybridoma (TIB-128, ATCC). The αCD11b Db was in the VH/VL orientation with the following general structure: VH-(G_4_S)-VL-Cys-H_6_ (Supporting Information S4). The αCD11b Db was produced in mammalian cells and purified by immobilized metal affinity chromatography (Ni-NTA) with good yields (2.1 g/L) and stored in an aqueous buffer (25 mM Tris-HCl, 1 mM NaCl, pH = 8.0). SDS-PAGE Gel confirmed that the diabody was the correct molecular weight (Figure S8).

### Synthesis of the CB-TE1K1P

DBCO-PEG4-CB-TE1K1P (CB-TE1K1P) was synthesized as previously described with minor modifications (Scheme S1).^10,31^ In brief, H-d-Lys­(Z)–OH (**7**) was treated with sodium nitrate, resulting in a diazonium salt which was then brominated using potassium bromide and hydrobromic acid. The resulting bromo carboxylic acid (**6**) was then benzyl protected using DCC­(*N*,*N*′-dicyclohexylcarbodiimide) and benzyl alcohol in the presence of DMAP­(4-Dimethylaminopyridine) to provide bromo benzyl ester (**5**). CB-cyclam was then alkylated with bromobenzyl ester (**5**) in the presence of potassium bicarbonate to give the monosubstituted CB-cyclam (**4**). Then, the resulting monosubstituted CB-cyclam (**4**) was reacted with formaldehyde and triethyl phosphite to afford diethyl phosphonate (**3**). Hydrogenation of CBz­(benzyloxycarbonyl) and benzyl ester, followed by ethyl ester hydrolysis, resulted in the primary amine (**2**). DBCO-PEG4-NHS was coupled to a primary amine (**2**) in the presence of triethylamine to provide the title compound (**1**). Final compound was determined to be >99% (Figure S9).

### Preparation of Azido-αCD11b Db

To provide a site of attachment for click chemistry on αCD11b Db, azidobutyric acid NHS ester was conjugated to the amino groups of the Db (Scheme 1). A stock solution of the azidobutyric acid NHS ester (10 mM) was prepared in DMSO. A portion of the azidobutyric acid NHS ester stock (4.5 μL, 45 nmol) was added to αCD11b Db (2 mg/mL,18 nmol) and allowed to react (37 °C, 2 h). The resulting reaction mixture was not purified, and this stock solution was used for subsequent click chemistry reactions.

### Preparation of AZdye488-αCD11b Db

To determine the substitution level of the azide conjugated to αCD11b Db by flow cytometry, AZdye488-αCD11b Db was generated. AZDye 488 DBCO (50 μg in 50 μL DMSO/H2O 1:1, 66 nmol) was added to a freshly prepared stock solution of azido-αCD11b Db (2 mg/mL,18 nmol). After coupling (37 °C,1.5 h), the reaction mixture was applied to a SEC preparative column (SRT-10 SEC-100 1.2 × 300 mm) and eluted with aqueous buffer (PBS, pH = 7.4), and the purification was monitored at 280 and 488 nm. The fractions containing the chelator-Db conjugate were pooled, then concentrated by centrifugal ultrafiltration (Thermo Scientific Pierce Protein Concentrators Pes, 10K MWCO), and stored at −80 °C. To assess the purity of the conjugate, SEC-HPLC was performed (Bio SEC-3, 4.6 mm × 300 mm, 280 and 488 nm). To determine the substitution level of azido groups on the Db, the absorbance (*A*_280 nm_ and *A*_497 nm,_ ND-1000 spectrophotometer, Thermo Scientific) was recorded to determine the substitution level.^13^

### Preparation of CB-TE1K1P-αCD11b Db

To generate CB-TE1K1P-αCD11b Db for radiolabeling, DBCO-PEG4-CB-TE1K1P (50 μg in 20 μL of PBS, 51 nmol) was added a freshly prepared stock solution of azido-αCD11b Db (2 mg/mL,18 nmol). After coupling (37 °C, 1.5 h), the reaction mixture was applied to a SEC preparative column (SRT-10 SEC-100 1.2 × 300 mm) and eluted with aqueous buffer (0.1 M NH_4_OAc, pH 5.5), and the purification was monitored by UV (280 nm). The fractions containing the chelator-Db conjugate were pooled, concentrated by centrifugal ultrafiltration (Thermo Scientific Pierce Protein Concentrators Pes, 10K MWCO), and stored at −80 °C. To assess the purity of the conjugate, SEC-HPLC was performed (Bio SEC-3 4.6 mm × 300 mm, monitored at 280 nm), which was calibrated with molecular weight markers.

### Radiolabeling

To generate [^64^Cu]­Cu-αCD11b Db, [^64^Cu]­CuCl_2_ (74 MBq) was added to a labeling buffer (0.05 mL of 0.1 M NH4OAc, pH 5.5) (Scheme 1B). After 5 min, CB-TE1K1P-αCD11b Db (8.5 nmol, 0.1 M NH4OAc) was added to the mixture for radiolabeling (2 h, 37 °C). The procedure of the reaction was monitored by iTLC. The [^64^Cu]­Cu-αCD11b Db radiotracer was diluted with unlabeled αCD11b Db to reach different specific activities. Final radiochemical purity was ≥95%, assessed with iTLC and SEC-HPLC, with molar activities that ranged from 1.06 to 8.51 MBq/nmol.

### Thermal Stability Assay

The thermal stability of Db AZdye488-αCD11b was assessed by flow cytometry. To induce thermal instability, AZdye488-αCD11b Db (100 nM, PBS) was heated (37 °C, 550 rpm orbital shaker), and at specific time points (0, 1, 2, 4, and 24 h), the samples were cooled (4 °C). Then, the thermally treated AZdye488-αCD11b Db samples were diluted (50 nM, PBS) with CD11b-positive RAW 264.7 cell lines (1 × 10^6^, preincubated with Ghost dye red,1.0 μg/1000 μL and washed with PBS twice, *n* = 3). Then, the samples were incubated at 37 °C for 30 min and washed with PBS twice. Additionally, the blocking test (with 100 mM αCD11b Db) was performed in parallel with the same conditions mentioned above. The cells were analyzed by four parameters (FSC, SCC, APC, and FITC). For each sample, 10,000 events were recorded at related gating areas. The fraction means fluorescence intensity (MFI) was calculated for each time point.

### Mouse Model of GBM

GL261 glioma cells were cultured in complete DMEM.^6^ Shortly before stereotactic injection, GL261 cells were resuspended in sterile DPBS at a concentration of 50,000 cells/μL. All animal studies were conducted under protocols approved by the University of Pittsburgh Institutional Animal Care and Use Committee (IACUC). For GBM tumor inoculation, C57BL/6j mice (female, 5–8 weeks old; Jackson Laboratories, Bar Harbor, ME) were anesthetized by isoflurane and placed on a stereotactic frame (Kopf Instruments). 1 × 10^5^ GL261 cells (1 × 10^5^) in 2 μL of DPBS were injected at a coordinate from the skull position of bregma of +2.5 mm ML, and −3.0 mm DV, using a micropump injector (World Precision Instruments). The mice received the radiotracer by intravenous injection in the tail vein at14 days after tumor inoculation.

### Immunoreactivity Assay

RAW 264.7 cell lines were resuspended in PBS, and 1 × 10^7^ cells were aliquoted in triplicates and about 50,000 CPM [^64^Cu]­Cu-αCD11b Db was added to each sample. The total number of CD11b receptors is about 10-fold relative to the added molar amount of [^64^Cu]­Cu-αCD11b Db. Samples were then incubated for 30 min at room temperature and centrifuged at 1500 rpm for 5 min for 3 times to wash away the supernatant. The CPM of cell pellets was counted and compared to the same amount of radiotracer (∼ 50,000 CPM) that was added into each sample to determine the immunoreactivity percentage.

To competitively inhibit tracer binding, the procedure of the blocking group was the same as that of the testing group, except that a 100-fold excess amount of αCD11b IgG (16.6 nmol, relative to CD11b receptors) was added into RAW 264.7 cell suspension as a blocking reagent before adding the radiotracer.

### *In Vivo* Imaging and Biodistribution Assessment

For PET/CT imaging, mice were injected intravenously with [^64^Cu]­Cu-αCD11b Db at differing molar activities (3.7 MBq, 8.51, 4.26, 2.12, and 1.06 MBq/nmol for groups 1 to 4). Mice were anesthetized with 2% isoflurane, and static PET/CT imaging was performed at 4 and 24 h. Images were coregistered and analyzed using VivoQuant 2021 and Imalytics 3.0 software. The regions of interest were drawn with guide by PET and CT signals. The radiotracer uptake was presented as SUV_mean_. Biodistribution studies of the radiotracer were performed in the same PET imaging cohort after 24 h of PET/CT imaging. The mice were euthanized after PET/CT imaging, the major organs were collected and weighed, and the tissue-associated radioactivity was recorded as CPMs in a gamma counter.

### Flow Cytometry: Determination of the Equilibrium Dissociation Constant

To determine the equilibrium dissociation constant, a nonbinding control for nonspecific binding was achieved with excess amount of αCD11b Db (100 mM per well).

To determine the equilibrium dissociation constant, resuspended RAW 264.7 cells (1 × 10^6^ cells per sample) were preincubated with Ghost dye red (1.0 μg, 1000 μL, DPBS, 15 min, 4 °C). The stained cells were washed twice with DPBS and divided into two groups. Group A was then incubated with AZdye488-αCD11b Db (concentration: 0.5, 1, 2, 5, 50, 100, 500, and 1000 nM) for 30 min at 4 °C. The stained cells were washed twice with DPBS, along with the unstained samples, for cytometric analysis. To acquire the data for nonspecific binding, additional Ghost Dye Red prestained cells were incubated with 100 mM αCD11b Db for 30 min. The cells were then incubated with different amounts of AZdye488-αCD11b Db under the same conditions described above. The cells were analyzed by four parameters (FSC, SCC, APC, and FITC). For each sample, 10,000 events were recorded at related gating areas. The resulting curves were fitted with GraphPad Prism 10 using a nonlinear regression model. The assay was repeated 3 times to generate SD.

### Imaging Figure of Merit (IFOM)

To determine the optimal imaging time for tumor-to-blood maximum, postadministration of [^64^Cu]­Cu-αCD11b Db, IFOM analysis was performed on PET imaging data.^11^ Six mice were injected with [^64^Cu]­Cu-αCD11b Db radiotracer with a molar activity of 8.51 MBq/nmol. The dose was 25 μg per mouse. The PET imaging data and SUV_mean_ of related organs and tumor tissue were collected at 2, 4, 6, 18, and 24 h. Since the SUV_mean_ of blood was difficult to obtain in PET images, the uptake of the area of heart chambers was determined as the uptake of blood. SUV_mean_ of the tumor was used as it is from PET imaging data.

### Statistical Analysis

Statistical analyses were performed using GraphPad Prism 9 and GraphPad Prism 10. Right-brain (tumor) and left-brain (contralateral control) SUV_mean_ and biodistribution were examined using an unpaired *t* test and two-way ANOVA. All tests were two-sided; a *p*-value of <0.05 is considered significant. Error values are reported as standard deviation (SD).

## Supplementary Material



## Notes

PET scans, SUV analysis, maximum intensity projections (MIPs), SDS-PAGE gel results, sequence of αCD11b Db, and synthesis data of DBCO-PEG4-CB-TE1K1P (PDF)
