# Supplementary material for: [64Cu]Cu-Labeled αCD11b Diabody as a Novel PET Tracer for the Detection of Immunosuppression in Glioblastoma
Source: ACS Omega. 2026 Feb 19;11(8):13835–44. doi: 10.1021/acsomega.5c11942 (PMC12961533; doi:10.1021/acsomega.5c11942)
Supplement: Supplementary file 1 [file ao5c11942_si_001.pdf]

[^{64}Cu]Cu-labeled αCD11b diabody as a novel PET tracer for detection of immuno-suppression in glioblastoma

Bo Li[1], Sydney A. Jackson[2], Amelia Stepniak[2], Peggy Birikorang[1], Dominic Menendez[1], Robert Edinger[4], Michael Pun[5], Charles M. Laymon[3], Carolyn J. Anderson[5,*], Gary Kohanbash[2,*], W. Barry Edwards[1,*]

[1]Department of Biochemistry, University of Missouri, Columbia, MO 65211, USA
[2]Department of Neurological Surgery, UPMC Children's Hospital of Pittsburgh, University of Pittsburgh School of Medicine, Pittsburgh, PA 15224, USA
[3]Department of Radiology, University of Pittsburgh School of Medicine, Pittsburgh, PA 15224, USA
[4]Department of Radiation Oncology, University of Pittsburgh, Pittsburgh, PA 15224, USA
[5]Department of Radiology, University of Missouri, Columbia, MO, 65211, USA
*Co-corresponding authors
Corresponding Authors:
W. Barry. Edwards, Ph.D.
Phone: +1 (573) 882-4845
E-mail: wbe59z@missouri.edu
Gary Kohanbash, Ph.D.
Phone: +1 (412) 692-9456
Email: gary.kohanbash2@chp.edu
Carolyn J. Anderson, Ph.D.
Phone: +1 (573) 882-6948
Email: carolyn.j.anderson@missouri.edu

Supplementary data

Contents

S1 PET scans and SUV analysis....................3
S2 MIPs of 64Cu-aCD11b Db in tumor-bearing mice....................6
S3 SDS-PAGE gel of αCD11b DB....................9
S4 Sequence of αCD11b DB....................10
S5 Synthesis of DBCO-PEG4-CB-TE1K1P....................11

S1 PET scans and SUV analysis

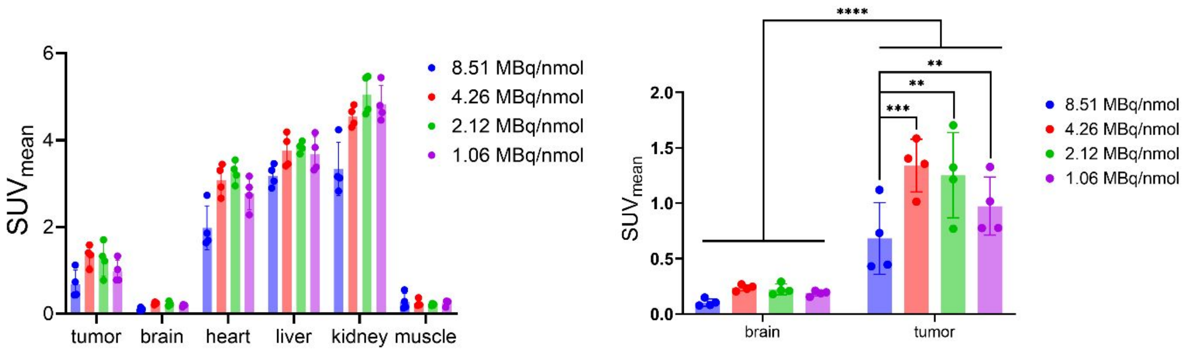

Figure S1. SUV$_{mean}$ of mice which received [^{64}Cu]Cu-αCD11b Db (4 h, post injection, 8.51, 4.26, 2.12 and 1.06 MBq/nmol). Right: Close-up data of SUV$_{mean}$ at brain and tumor tissue.

.

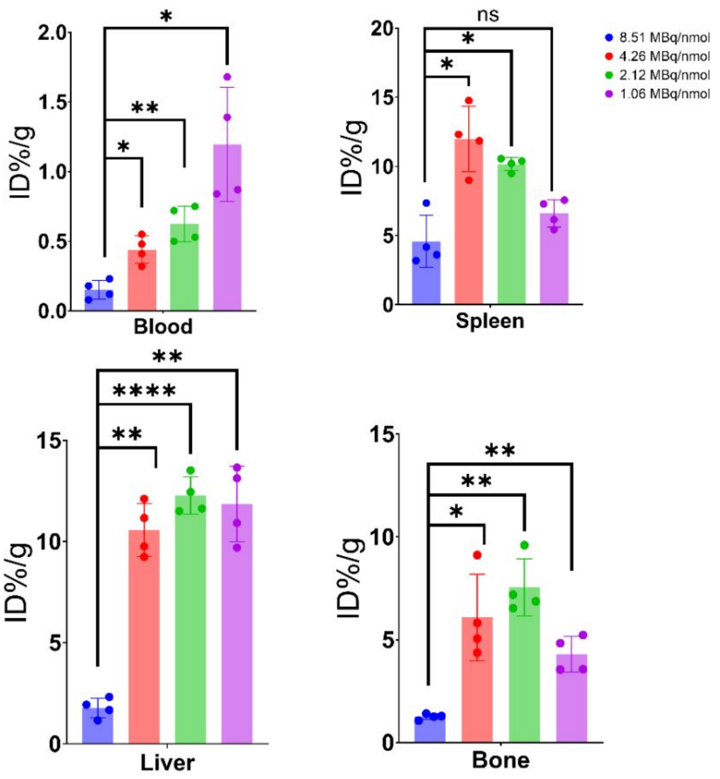

Figure S2. Biodistribution of [^{64}Cu]Cu-αCD11b Db of varying molar activities at 24 hours post injection.

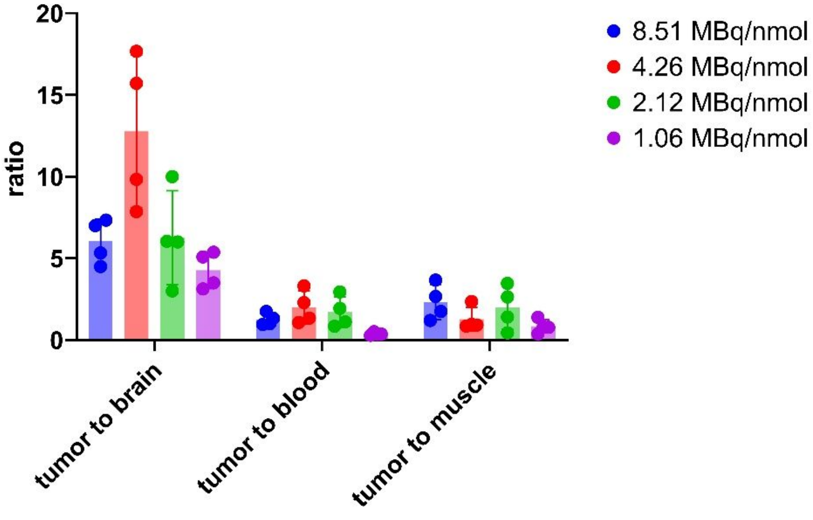

Figure S3. The ratio of SUV_{mean} of tumor to selected organs activities of [^{64}Cu]Cu-αCD11b Db with varying molar activities at 4 hours post injection.

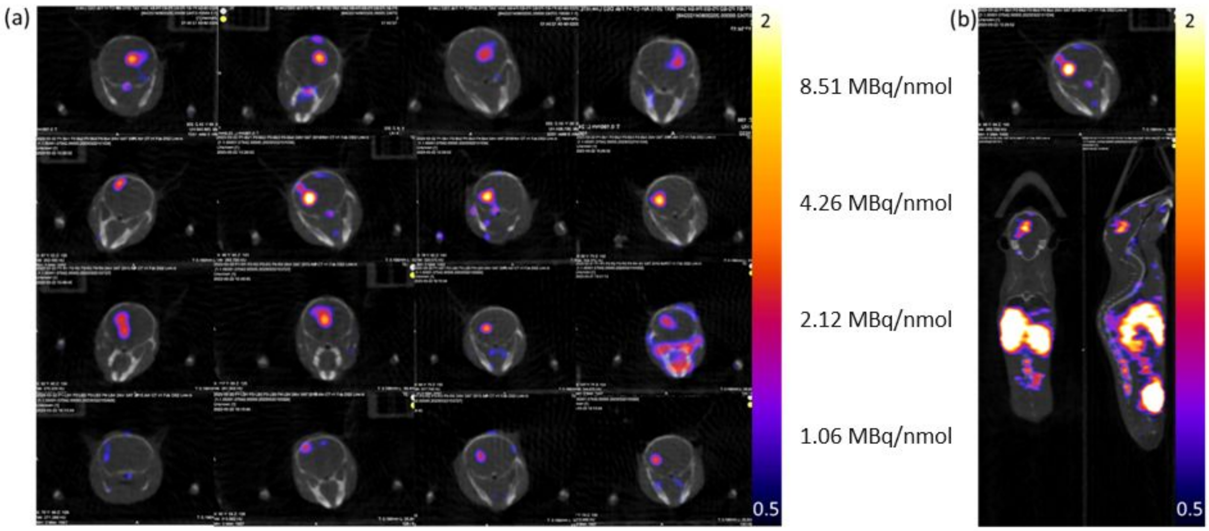

Figure S4. (a) Axial PET/CT of tumor bearing mice (n=4) which received [^{64}Cu]Cu-αCD11b Db of varying molar activity (4 h, post injection, 8.51, 4.26, 2.12 and 1.06 MBq/nmol). (b) PET/CT of a tumor bearing mouse with axial, frontal and sagittal views (4 h post injection, 4.26 MBq/nmol . The color bar represents SUV.

S2 MIPs of 64Cu-aCD11b Db in tumor-bearing mice

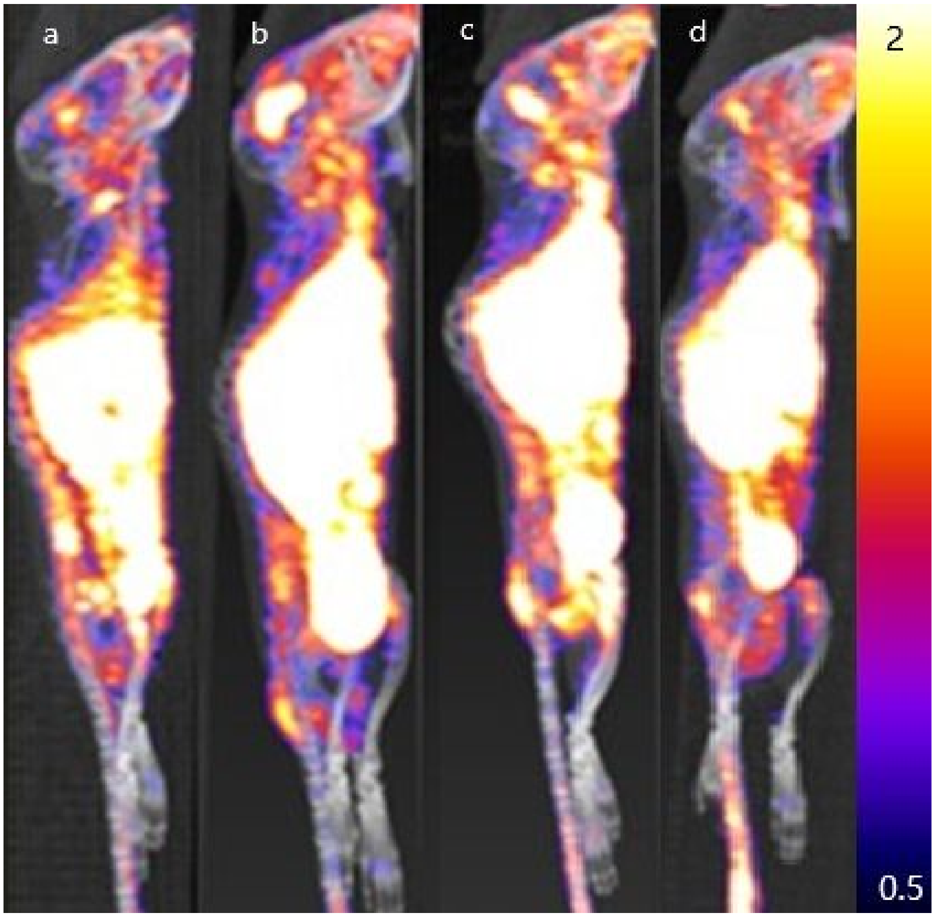

Figure S5. Selected MIPs of the PET/CT of tumor bearing mice (4 h post injection, (a) 8.51, (b) 4.26, (c) 2.12 and (d) 1.06 MBq/nmol). The color bar represents SUV.

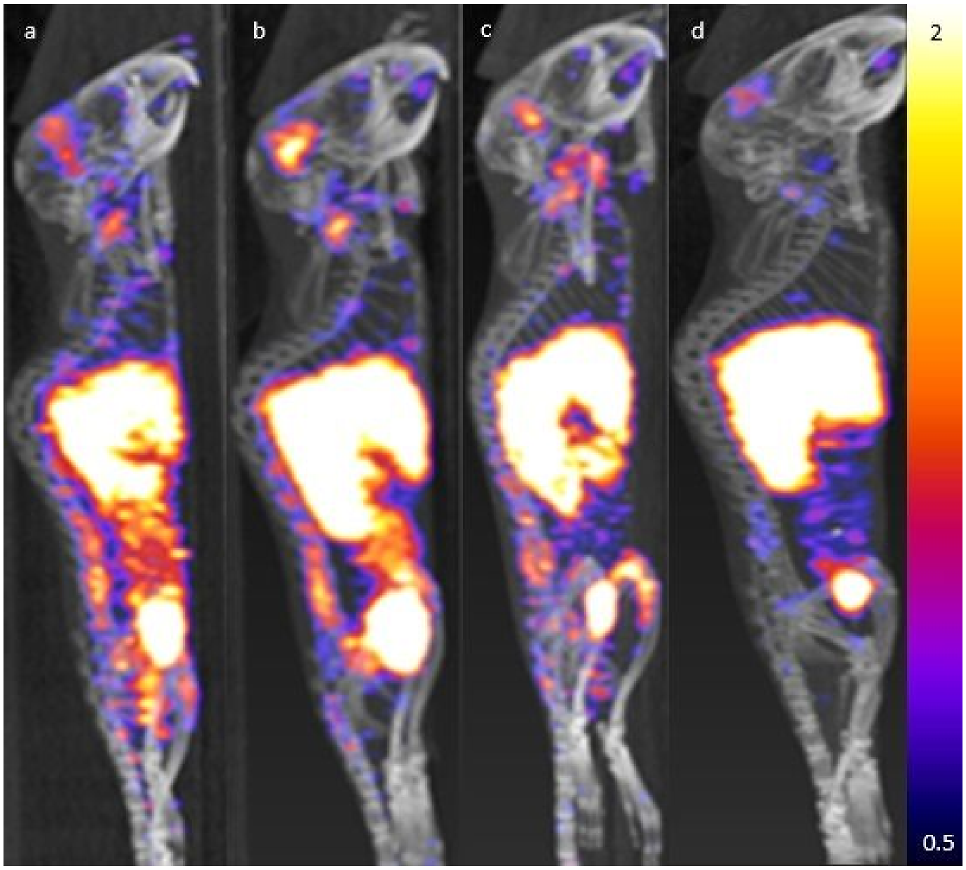

Figure S6. Selected MIPs of the PET/CT of tumor bearing mice (24 h post injection, (a) 8.51, (b) 4.26, (c) 2.12 and (d) 1.06 MBq/nmol). The color bar represents SUV.

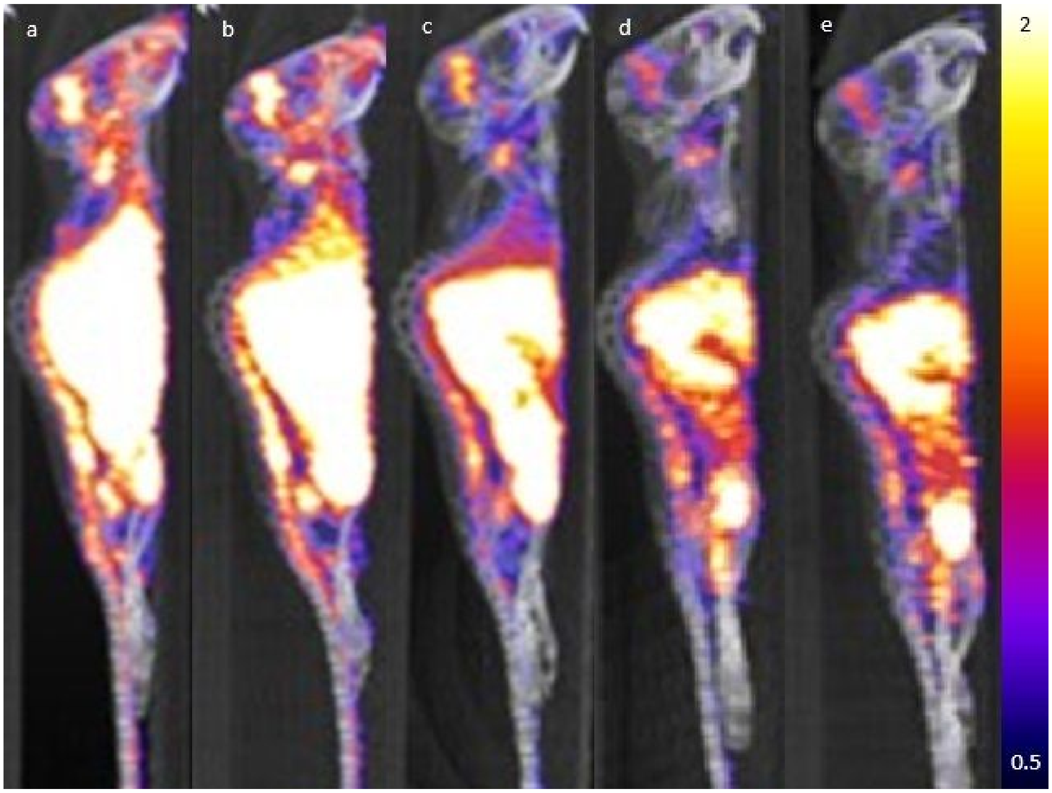

Figure S7. Selected MIPs of the PET/CT of tumor bearing mice (8.51 MBq/nmol for each, (a) 2h, (b) 4h, (c) 6h, (d) 18h and (e)24h post injection). The color bar represents SUV.

S3 SDS-PAGE gel of αCD11b DB

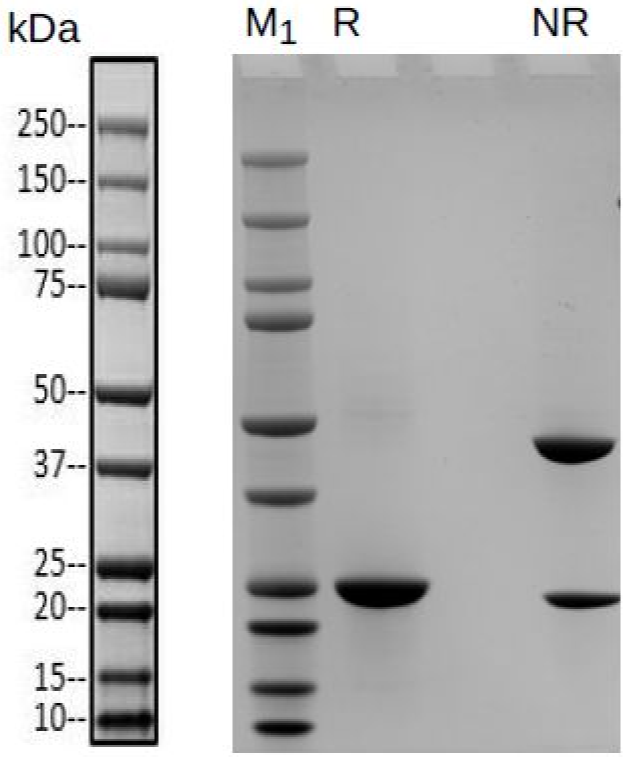

Figure S8. The SDS-PAGE gel data of the αCD11b Db. M1 refers to protein markers. R and NR refer to reducing and non-reducing conditions.

S4 Sequence of αCD11b DB

QVQLKESGPGLVQPSQTLSLTCTVSGFSLTSNSISWVRQPPGKGLEWMGAIWSGGGTDYNSD
LKSRLSIIRDTSKSQVFLKMNSLQTDDTAIYFCTRGGYPYYFDYWGQGVMVTVSSGGGGSDIV
MTQSPSSLAVSAGETVTINCKSSQSLLYSENQENYLAWYQQKPGQSPKLLIYWASTRQSGVPD
RFIGSGSGTDFTLTISSVQAEDLAIYYCQQYYDTPLTFGSGTKLEIKAAHHHHHHGGC

S5 Synthesis of DBCO-PEG4-CB-TE1K1P

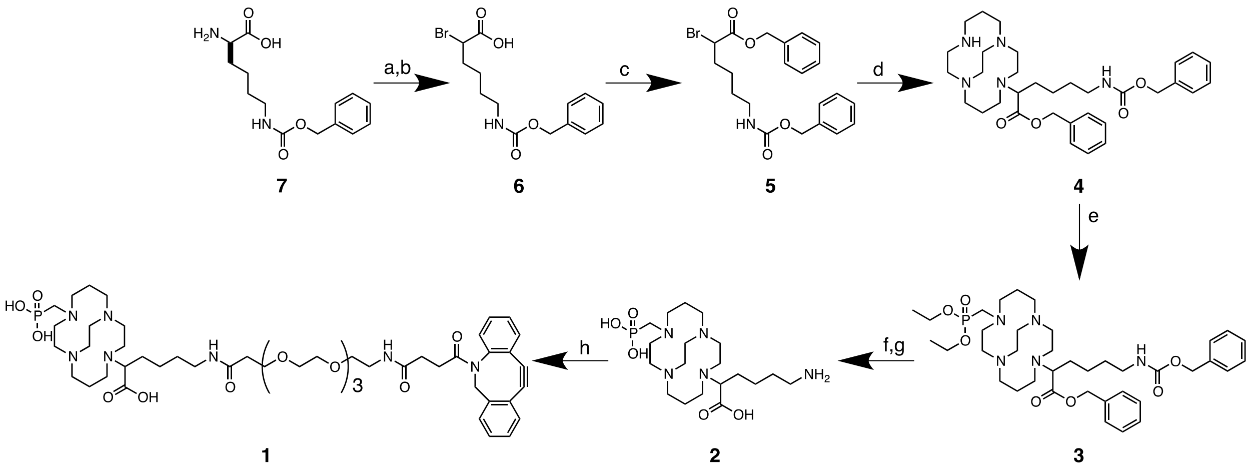

Scheme (S1). Synthesis of DBCO-PEG4-CB-TE1K1P. a) $NaNO_2$, b) KBr/HBr, 0°C 2h. c) DCC, BnOH, DMAP, RT 8h. d) CB-Cyclam, $KHCO_3$, ACN, RT, 24h. e) triethyl phosphite, formaldehyde, THF, RT, 24h. f) H_2, Pd/C. g) 6M HCl, reflux 12h. h) DBCO-PEG4-NHS, TEA, DMF, RT 24h.

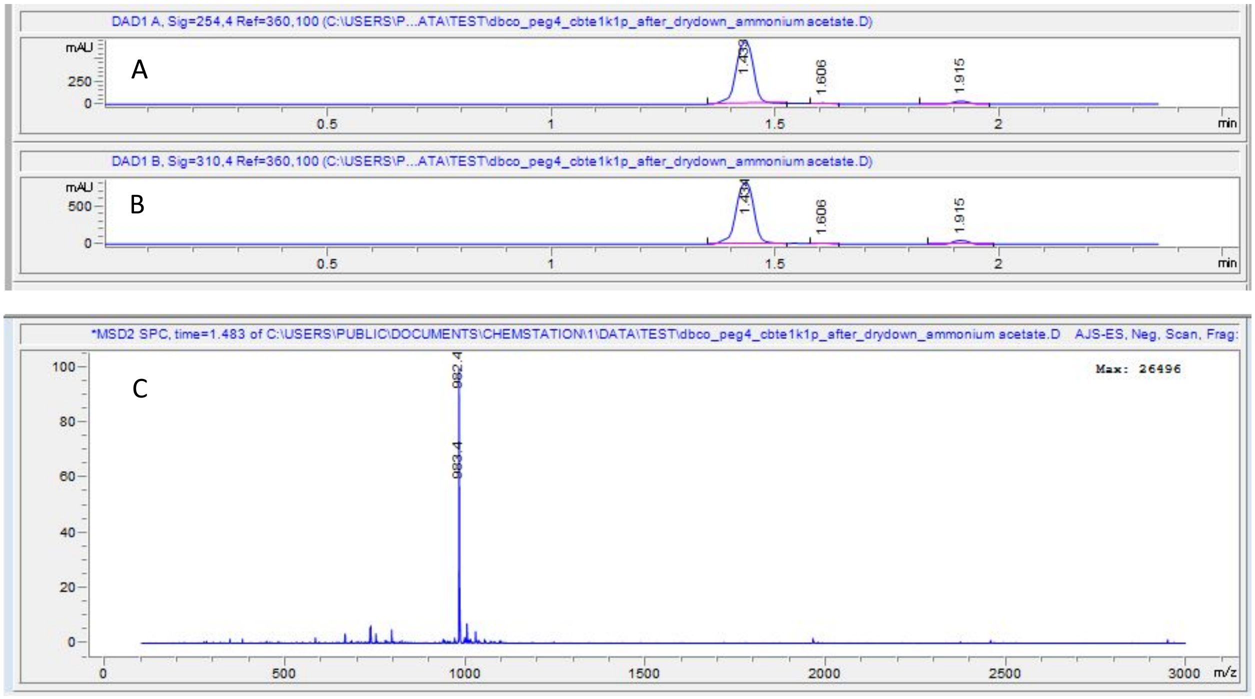

Figure (S9). LCMS characterization of compound 1. UV chromatograms at (A) 254 nm and (B) 310 nm. (C) extracted mass spectra [M-H] calculated = 982.5. Observed [M-1] = 982.4
